# Standardizing Point-of-Care Ultrasound (POCUS) Credentialing in Internal Medicine Training

**DOI:** 10.7759/cureus.92215

**Published:** 2025-09-13

**Authors:** Apurva Popat, Muhammad Haseeb Zubair, Ateeq U Rehman

**Affiliations:** 1 Cardiology, Marshfield Clinic Health System, Marshfield, USA; 2 Anesthesiology, Marshfield Clinic Health System, Marshfield, USA; 3 Internal Medicine, Marshfield Clinic Health System, Marshfield, USA

**Keywords:** cardiac pocus, internal medicine (general medicine), medical residency, pocus echocardiogram, point of care ultrasound (pocus)

## Abstract

Point-of-care ultrasound (POCUS) is increasingly recognized as a valuable tool in internal medicine for enhancing physical examination and improving diagnostic accuracy. Growing evidence shows that integrating POCUS into routine care improves diagnostic accuracy, shortens time to diagnosis, increases resident confidence, and enhances patient safety by reducing procedure-related complications. However, POCUS training remains inconsistent across internal medicine residency programs, with no national requirement for education or competency - unlike emergency medicine, which has established minimum scan requirements. To address this gap, we propose “POCUS Championship Pathway,” a longitudinal curriculum integrated across all three years of residency. Interns begin with a structured bootcamp covering ultrasound fundamentals, followed by continued learning through conferences, hands-on sessions, and supervised scanning during routine inpatient and ICU rounds. A two-week “POCUS Deep Dive” elective is also offered for senior residents requiring submission of reviewed ultrasound clips across five key domains - cardiac, lungs/pleura, abdomen, lower-extremity deep vein thrombosis (DVT), and soft tissue - with successful completion recognized by a certificate and enabling them to apply for credentialing. We propose that all internal medicine residency programs should consider adopting a structured POCUS curriculum, and the Accreditation Council for Graduate Medical Education (ACGME) committee may consider establishing formal requirements to ensure consistent training and competency nationwide.

## Editorial

Internal medicine residents increasingly recognize the clinical value of point-of-care ultrasound (POCUS) in enhancing the physical exam and expediting diagnosis. Despite broad recognition, POCUS training remains highly variable across internal medicine programs and amongst hospitalists [1]. By 2019, the Alliance for Academic Internal Medicine (AAIM) had already noted that POCUS “has shown clinical benefit to patient care within internal medicine” and urged integration of POCUS across the training spectrum [2]. Across the country, no national mandate or accrediting requirement compels internal medicine residencies to teach POCUS or certify competence. On the other hand, emergency medicine mandates logging at least 150 scans per resident [3]. Consequently, interested residents at one hospital may graduate fluent in bedside ultrasound, while others finish training knowing little beyond landmark anatomy [4]. We suggest that all internal medicine residencies consider adopting structured POCUS credentialing, as adopted by Marshfield Clinic Health System (MCHS) in their internal medicine longitudinal POCUS training program, i.e., the “POCUS Championship Pathway" [5].

**Figure 1:**
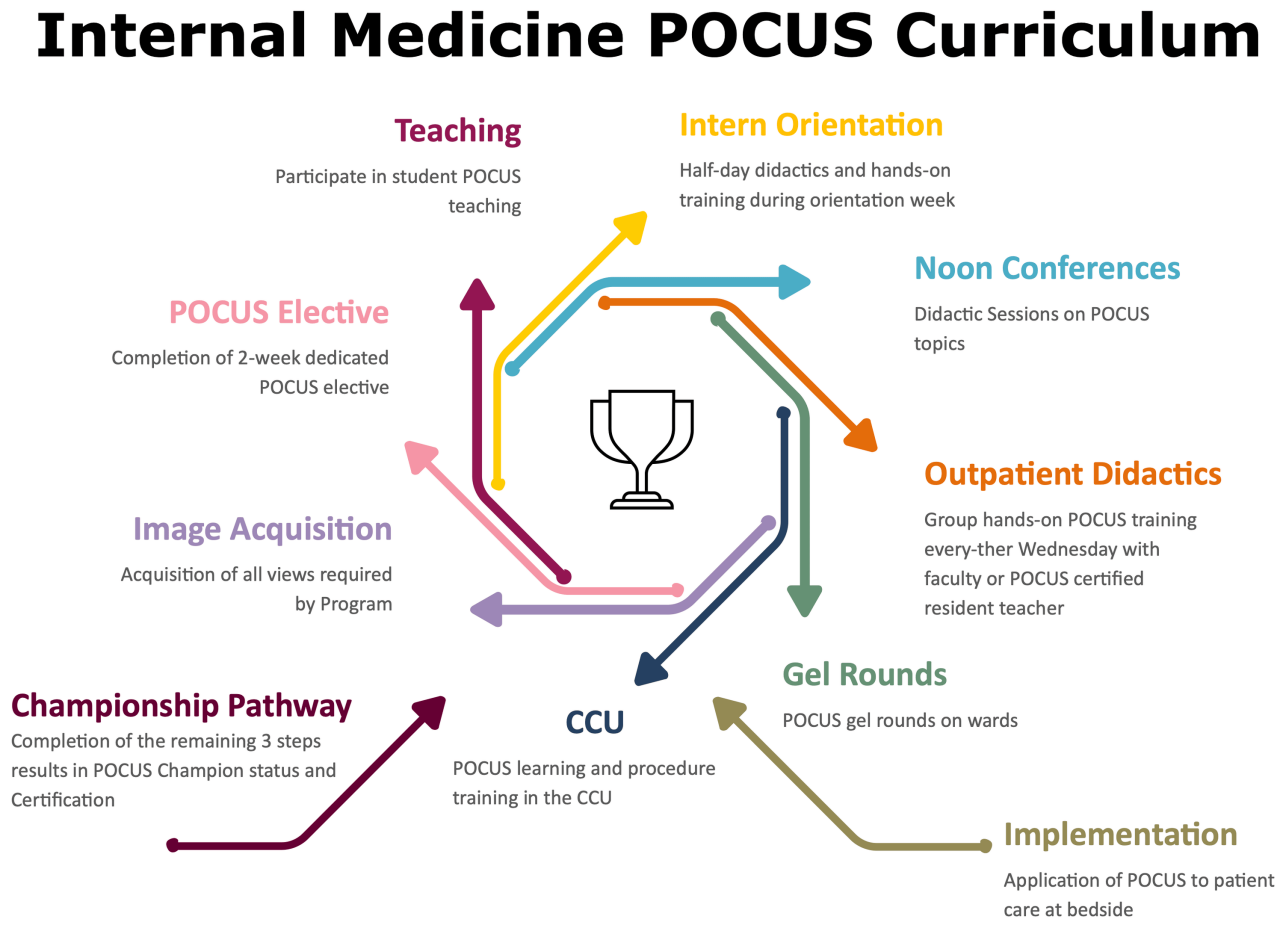
Longitudinal internal medicine POCUS curriculum This diagram outlines the stepwise longitudinal curriculum designed to build progressive ultrasound competency across all three years of internal medicine residency. Training begins with a half-day simulation and didactic session during intern orientation, followed by recurring noon conferences, outpatient hands-on sessions, and daily “gel rounds” in inpatient wards and the ICU. Residents participate in structured image acquisition, complete a dedicated two-week POCUS elective, engage in peer teaching, and apply ultrasound in direct patient care. Completion of all components qualifies residents for the "POCUS Champion" designation and institutional certification. POCUS: Point-of-care ultrasound; CCU: Coronary care unit Figure credits: Marshfield Clinic POCUS Curriculum. © Marshfield Clinic, Inc. All rights reserved. Used with permission from Marshfield Clinic, Inc., Marshfield, WI, USA [5].

The "POCUS Championship Pathway" emphasizes that a brief elective alone is insufficient; instead, ultrasound education should be woven through the entire three-year residency (Figure 1). At MCHS, new interns first participate in a half-day session combining didactics and simulation-based training. This bootcamp introduces ultrasound fundamentals (machine usage, knobology, and imaging basics) and core applications relevant to internal medicine. Interns at this point are taught to ask specific questions, attempt to answer them using a quick bedside ultrasound, and correlate clinically with the overall picture. By front-loading these skills, interns enter the wards with a baseline ability to obtain images. Formal POCUS instruction continues throughout residency via conferences and workshops. In the outpatient setting, residents also attend dedicated hands-on ultrasound sessions. During these sessions, small groups practice scanning under supervision, led by a faculty mentor or a senior resident who has earned a POCUS teaching credential. In the inpatient ward, teams are encouraged to perform “gel rounds,” incorporating bedside ultrasound exams into their routine rounding process. In the medical ICU, this practice extends to critical care rounds as well - residents use POCUS for rapid assessment of unstable patients and to guide procedures. Attending physicians with critical care ultrasound expertise provide oversight and real-time feedback. For easy access, all four teams in inpatient rotation have their own handheld ultrasound devices, in addition to ultrasound machines available in the medical ICU and the emergency room. The goal of our model is to encourage residents to use POCUS every day and appreciate normal and abnormal findings.

Availability of handheld ultrasound devices varies greatly across internal medicine residencies and mostly depends on local finances and Graduate Medical Education (GME) educational support. In our program, the Department of Education (via GME funds) purchases the devices. We have four floor call teams, and each team has one handheld device that the senior, the intern, and the attending on that team use, which works out to about one device per two residents on service. The senior is responsible for the device and the night handoff; at sign-out, the device is passed to the night team and stored in the team call room on a charging dock. The typical purchase cost is about $4,000-$5,000 per device. We are not proposing a universal ratio, but one device per ward team has been practical and reliable for us.

We also offer a structured two-week "POCUS Deep Dive" elective for postgraduate year (PGY)-2 and PGY-3 residents [5]. Residents are required to submit high-quality ultrasound video clips across five key domains, including cardiac, lungs, abdomen, deep vein thrombosis (DVT), and soft tissue ultrasound. The complete list of required image sets and minimum numbers is detailed in Table 1. These archived studies are reviewed by POCUS faculty for quality and accuracy. Residents who meet the image and interpretation criteria receive a certificate of completion, which can support future hospital POCUS credentialing applications.

**Table 1:** Required POCUS image sets for credentialing in the Marshfield Clinic internal medicine elective POCUS: Point-of-care ultrasound; DVT: Deep vein thrombosis; RLQ: Right lower quadrant; LLQ: Left lower quadrant; CFV: Common femoral vein; PLAX: Parasternal long-axis; PSAX: Parasternal short-axis; IVC: Inferior vena cava; A4C: Apical four-chamber Table credits: Created by the authors.

System	View/Structure	Number of Images
Cardiac	PLAX	10
	PSAX at mid-ventricle/papillary level	10
	A4C	10
	Subcostal four-chamber	10
	IVC - longitudinal	10
Lungs and pleura	Normal sliding with A-lines	3
	Consolidation (suspected pneumonia)	3
	Pleural effusion	3
	B-lines (≥1 B-line)	3
Abdomen	Right kidney with hepatorenal recess	3
	Left kidney with splenorenal space	3
	Abdominal aorta - transverse	3
	Abdominal aorta - longitudinal	3
	Bladder - transverse	3
	Bladder - longitudinal	3
	Ascites in the RLQ or LLQ	3
Lower extremity DVT	Right common femoral vein	3
	Right CFV - greater saphenous vein junction	3
	Right femoral vein - deep femoral junction	3
	Right mid/distal femoral vein	3
	Right popliteal vein	3
	Left common femoral vein	3
	Left CFV - greater saphenous vein junction	3
	Left femoral vein - deep femoral junction	3
	Left mid/distal femoral vein	3
	Left popliteal vein	3
Soft tissue	Normal subcutaneous tissue	2
	Subcutaneous edema (“cobble-stoning”)	2

Longitudinal POCUS training yields substantial and sustained gains in resident knowledge, technical proficiency, and clinical confidence. The American College of Physicians (ACP) evidence report concluded that adding POCUS to standard diagnostic pathways results in a statistically significant increase in correct diagnoses compared to standard care alone, with improvements observed across conditions such as congestive heart failure, pneumonia, pulmonary embolism, pleural effusion, and pneumothorax [6]. Procedural safety is another critical benefit. POCUS guidance for procedures such as central venous catheterization, thoracentesis, and paracentesis reduces complication rates, lowers associated costs, and shortens hospital stays related to procedural adverse events [7]. These benefits are directly linked to the operator’s skill and experience, which are best developed and maintained through longitudinal, structured training rather than isolated workshops.

Successful implementation of longitudinal POCUS curricula requires a multifaceted approach, addressing common barriers such as a lack of trained faculty, limited access to equipment, and the absence of standardized national guidelines. Programs have overcome these barriers through creative use of external resources, such as national POCUS certificate programs, peer-to-peer teaching, and interdepartmental collaboration. For example, a university-based internal medicine residency program leveraged a national POCUS certificate program and local peer teaching to deliver longitudinal training despite limited local faculty expertise, resulting in increased resident use and comfort with both diagnostic and procedural POCUS applications [8]. Faculty development is critical for sustainability and expansion. Programs should invest in longitudinal, hands-on faculty training with ongoing supervision and feedback, integration of POCUS into clinical and educational workflows, and the cultivation of a supportive institutional culture [9].

In conclusion, POCUS has reached a turning point in internal medicine. Some trainees finish residency with advanced POCUS skills, while others graduate having never held a probe. Making POCUS an Accreditation Council for Graduate Medical Education (ACGME) requirement urgently would drive universal adoption, align curricula around core competencies with supervised scan portfolios and quality assurance, and simplify downstream hospital credentialing - improving diagnostic accuracy, resident confidence, and patient safety.
